# Democratizing protein language models with parameter-efficient fine-tuning

**DOI:** 10.1073/pnas.2405840121

**Published:** 2024-06-20

**Authors:** Samuel Sledzieski, Meghana Kshirsagar, Minkyung Baek, Rahul Dodhia, Juan Lavista Ferres, Bonnie Berger

**Affiliations:** ^a^AI for Good Research Lab, Microsoft Corporation, Redmond, WA 98052; ^b^Computer Science and Artificial Intelligence Laboratory, Massachusetts Institute of Technology, Cambridge, MA 02139; ^c^Department of Biological Sciences, Seoul National University, Seoul 08826, South Korea; ^d^Department of Mathematics, Massachusetts Institute of Technology, Cambridge, MA 02139

**Keywords:** protein language models, parameter-efficient fine-tuning, protein–protein interactions, homooligomer symmetry, quaternary structure

## Abstract

Proteomics has been revolutionized by large protein language models (PLMs), which learn unsupervised representations from large corpora of sequences. These models are typically fine-tuned in a supervised setting to adapt the model to specific downstream tasks. However, the computational and memory footprint of fine-tuning (FT) large PLMs presents a barrier for many research groups with limited computational resources. Natural language processing has seen a similar explosion in the size of models, where these challenges have been addressed by methods for parameter-efficient fine-tuning (PEFT). In this work, we introduce this paradigm to proteomics through leveraging the parameter-efficient method LoRA and training new models for two important tasks: predicting protein–protein interactions (PPIs) and predicting the symmetry of homooligomer quaternary structures. We show that these approaches are competitive with traditional FT while requiring reduced memory and substantially fewer parameters. We additionally show that for the PPI prediction task, training only the classification head also remains competitive with full FT, using five orders of magnitude fewer parameters, and that each of these methods outperform state-of-the-art PPI prediction methods with substantially reduced compute. We further perform a comprehensive evaluation of the hyperparameter space, demonstrate that PEFT of PLMs is robust to variations in these hyperparameters, and elucidate where best practices for PEFT in proteomics differ from those in natural language processing. All our model adaptation and evaluation code is available open-source at https://github.com/microsoft/peft_proteomics. Thus, we provide a blueprint to democratize the power of PLM adaptation to groups with limited computational resources.

## Significance

While large machine learning models have had a transformative impact on computational modeling of proteins, the computation and memory requirements to train them remain a barrier to adoption for academic labs, biotech startups, and experimental labs with limited computational resources. Techniques for efficient, scalable training of such models would substantially increase their accessibility and adoption for a variety of proteomic tasks. We bring techniques for parameter-efficient fine-tuning (FT) from natural language processing to large protein language models, and demonstrate their efficacy as an alternative strategy to resource-intensive full FT for adapting to downstream tasks. We show that these methods remain competitive with significantly less computational cost and lay the groundwork for best practices for their use in proteomic modeling.

**Fig. 1.**
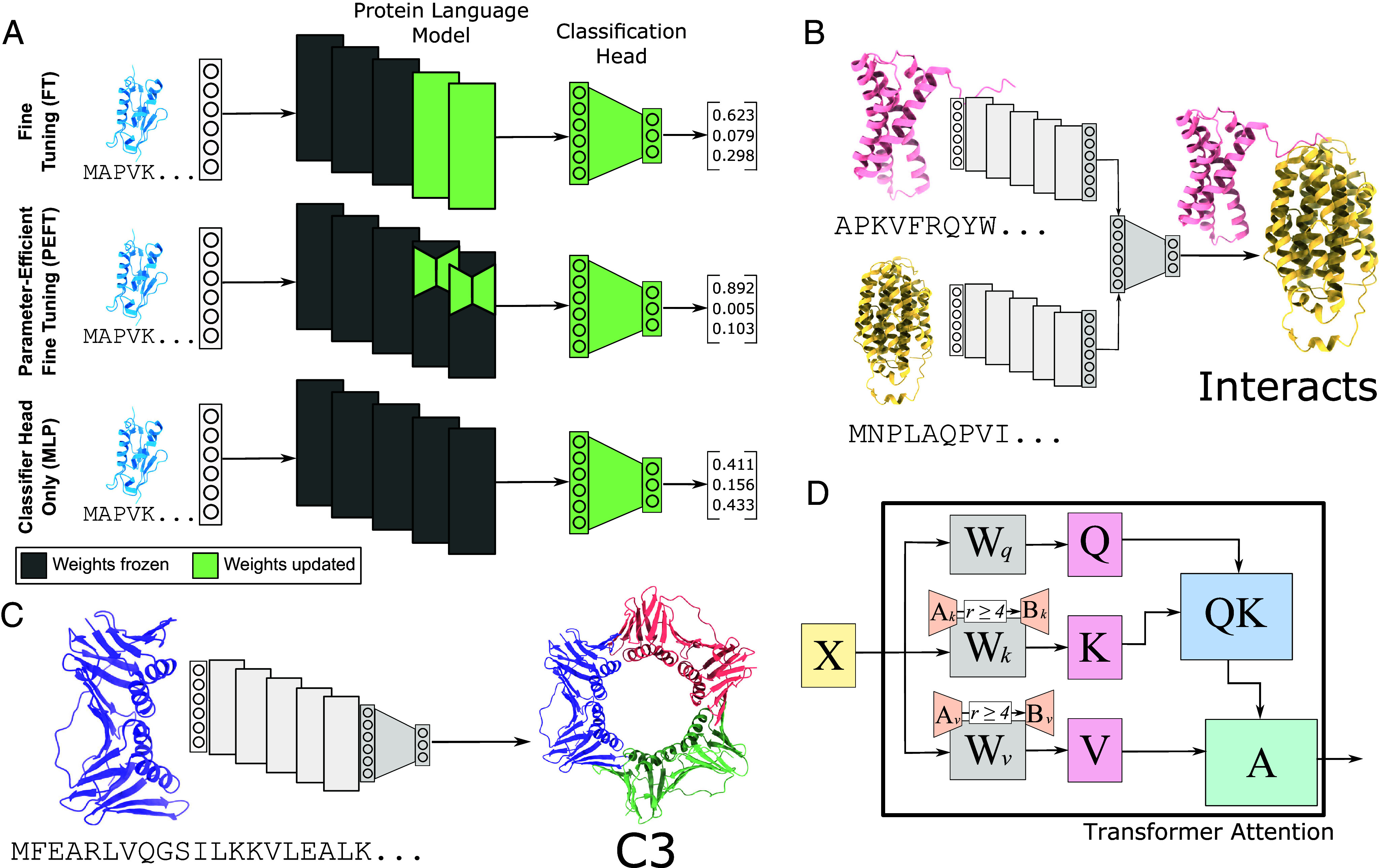
Bringing PEFT to proteomics. (*A*) The traditional paradigm for adapting PLMs to a specific downstream task is to fine-tune the parameters of the last *n* layers along with a new classification head (*Top*, FT). Here, we introduce PEFT to proteomics, tuning only the parameters of low-rank adapter matrices added to these final layers rather than the full weight matrices (*Middle*, PEFT). We compare also with a baseline that uses the embeddings as-is and trains only the classification head (*Bottom*, MLP). (*B*) We show that PEFT and MLP models achieve performance competitive with the state-of-the-art on predicting PPIs. (*C*) We also train PEFT models to predict the symmetry class of homooligomers, showing that the performance of PEFT models only slightly trails that of FT models yet uses several orders of magnitude fewer parameters. (*D*) We show the transformer self-attention with LoRA weight matrices added. We explore the hyperparameter space of low-rank adapters, creating a blueprint for best applying LoRA to PLMs. Notably, this blueprint differs from that in NLP. We recommend adding adapters with rank at least 4 to the key and value weight matrices of the self-attention layers. We explore different combinations of rank and the LoRA hyperparameter *α* in “Impact of LoRA Hyperparameters on Performance”. *X*: input to attention head. $W_{q,k,v}$: query, key, and value weights. $A_{k,v},B_{k,v}$: newly added low-rank adapter weights. Q,K,V: query, key, and value representations. *QK*: intermediate product of *Q* and *K*. *A*: output of attention layer.

The introduction of large pretrained protein language models (PLMs) has transformed the computational modeling of protein sequence, structure, and function. These models are trained in an unsupervised manner on tens or hundreds of thousands of protein sequences, and they learn hidden representations which contain information about evolutionary constraints, chemical properties, secondary structure, and more (1). These representations generalize broadly, which enables PLMs to be tuned to a wide variety of proteomic tasks. In the broader machine learning context, such large, pretrained, and task-agnostic models are referred to as “foundation” models. Typically, when a foundation model is tailored to a specific downstream task, the parameters of the pretrained model are updated in a process known as fine-tuning (FT), that adapts the parameters to fit the task-relevant supervised data (Fig. 1*A*, *Top*). However, as the size of foundation models increases, FT a model for a task of interest is increasingly computationally expensive, often with heavy GPU memory requirements. This puts such tuning out of the reach of many research groups, especially in academic, government, or startup environments. As increasingly large PLMs continue to be developed, such as the 6.4 billion parameter ProGen2 (2) or the 15 billion parameter ESM2 (3), the computational expense of FT will continue to be a pressing issue in proteomics.

Natural language processing (NLP) has seen a similar increase in foundation model size, with the largest NLP models approaching or exceeding one trillion parameters (4). To address this hurdle, one recent NLP approach to FT uses prompt tuning (5), which circumvents updating the model’s parameters altogether and instead updates additional input prompt embeddings, thus enabling the model to make “zero shot” predictions (6); such an approach has been translated to generating protein sequences with some success (7).

Here, we draw inspiration from alternative methods developed for parameter-efficient fine-tuning (PEFT) in NLP (see ref. 8 for a survey). These methods, usually focused on the transformer layers of natural language models, add a small number (typically <1% of total model size) of new parameters which are tuned, leaving the original model parameters untouched (9, 10). These approaches require significantly fewer resources and can surprisingly sometimes achieve performance comparable to traditional FT (8) of all parameters in the layer in NLP tasks.

In this work, we introduce PEFT methods to large PLMs, remarkably demonstrating performance competitive with or exceeding traditional FT using parameter-efficient training on two important proteomic tasks—homooligomer symmetry prediction and protein–protein interaction (PPI) prediction. Homooligomers (proteins that form complexes with copies of themselves) often adopt symmetric conformations, and predicting the symmetry that a homooligomer adopts is an important task in structural biology and protein design (11) (Fig. 1*C*). We show that as in NLP, a PEFT model slightly trails behind the performance of FT (AUPR=0.400 vs. 0.489) but significantly outperforms baselines with frozen embeddings (AUPR=0.238) and offers a much more compute-efficient alternative (Table 1).

**Table 1.** Applying PEFT to train models for homooligomer symmetry

	MLP	PEFT (12 layers)	FT (8 layers)
# Trainable params.	89,857	657,810	157,585,810
Accuracy	0.206	0.393	**0.413**
F1	0.228	0.405	**0.455**
MCC	0.299	**0.464**	0.460
AUPR	0.238	0.400	**0.489**
Precision	0.341	0.477	**0.618**
Recall	0.206	0.393	**0.413**
Specificity	0.960	**0.970**	**0.970**

We trained multiple variants of ESM2 to predict the symmetry of homooligomers from primary sequence. MLP indicates a multilayer perceptron trained on embeddings from a frozen model, while PEFT and FT indicate PEFT and traditional FT of the transformer layers. Due to the reduced memory footprint of PEFT, we were able to fine-tune four additional layers (12 vs. 8). While traditional FT still yields the best performance, PEFT is competitive across many metrics and significantly outperforms training only the classification head. A random classifier would be expected to have an AUPR ≈0.055.

We further explore how PEFT models perform in predicting general PPIs from primary sequence, an important and well-studied problem in proteomics (12–14) (Fig. 1*B*). Here too, we find that PEFT models are competitive with FT models (*AUPR* = 0:600 vs. 0.623). Intrigued by these findings, for both tasks we also tested the baseline model, which trains a multilayer perceptron (MLP) classifier on embeddings from a frozen language model (Fig. 1*A*, *Bottom*). While MLP performance lags behind on symmetry prediction, we surprisingly find that this method actually outperforms both tuning methods (AUPR=0.684) for PPI, demonstrating the continuing efficacy of simple downstream models in proteomics when PLM embeddings are used. In fact, we show that all three approaches for model adaptation outperform the current state-of-the-art on a gold-standard benchmark from Bernett et al. (15) on several metrics (Table 2).

**Table 2.** Applying PEFT to train models for PPI

	Best prior	MLP	PEFT (5 layers)	FT (4 layers)
# Trainable params.	-	88,769	368,897	78,873,857
Validation				
Accuracy	-	0.595	0.596	**0.597**
F1	-	0.576	**0.648**	0.612
MCC	-	-	**0.201**	0.194
AUPR	-	**0.632**	0.620	0.622
Precision	-	**0.603**	0.574	0.590
Recall	-	0.552	**0.742**	0.636
Specificity	-	-	0.450	**0.557**
Test				
Accuracy	0.56 (Topsy-Turvy)	**0.631**	0.608	0.604
F1	0.61 (SVM-PCA)	0.632	**0.666**	0.631
MCC	0.15 (Topsy-Turvy)	**0.261**	0.230	0.210
AUPR	-	**0.684**	0.600	0.623
Precision	**0.65** (Topsy-Turvy)	0.630	0.580	0.591
Recall	0.77 (SVM-PCA)	0.633	**0.780**	0.676
Specificity	**0.86** (Topsy-Turvy)	0.623	0.436	0.532

We trained multiple variants of ESM2 to predict PPIs, and evaluate using the benchmark datasets from Bernett et al. (15). MLP indicates a MLP trained on embeddings from a frozen model, while PEFT and FT indicate PEFT and traditional FT of the transformer layers. Due to the reduced memory footprint of PEFT, we were able to fine-tune an additional layer. Simply using ESM2 embeddings with an MLP classifier outperforms the best prior methods across most metrics. The PEFT model achieves increased recall and F1 score compared to the MLP model and also outperforms the best prior. Both MLP and PEFT are competitive with FT. These best-prior methods were trained and tested on the same splits we evaluate here. These data are balanced, thus a random classifier would be expected to have an accuracy/AUPR ≈0.5.

We create a blueprint for applying PEFT methods to PLMs on proteomics tasks by performing extensive experiments on LoRA hyperparameter choices (Fig. 1*D*). Contrary to what is recommended for NLP tasks, we find that adding LoRAs to only the key and value matrices of the transformer achieves optimal performance, and that performance drops off as the rank of adapter matrices drops below four (Table 3). Our work shows that it is possible to achieve competitive performance with significantly fewer resources than traditional approaches, opening up the power of PLM FT to academic labs, small biotech startups, and other research groups that lack substantial computational resources.

**Table 3.** Variation in rank is tolerated, but performance degrades at too low a rank

Rank	# Trainable params.	Val. AUPR	AUPR	Acc.	F1	MCC	Prec.	Rec.	Spec.
1	189,697	0.621	0.600	0.592	0.635	0.190	0.575	0.710	0.474
2	215,297	0.619	0.586	0.595	0.614	0.190	0.586	0.644	**0.545**
4	266,497	**0.644**	**0.633**	**0.604**	**0.658**	**0.219**	0.579	0.761	0.447
8	368,897	0.637	**0.633**	0.601	0.630	0.205	0.587	**0.680**	0.522
64	1,802,497	0.638	0.623	0.601	0.630	0.205	**0.588**	0.679	0.523

Hu et al. (9) show that LoRA is robust to rank *r* values as low as one. We test whether this robustness holds for protein language and on the PPI prediction task. Test set performance remains relatively strong across all ranks tested and actually peaks at *r* = 4, but there is a noticeable drop-off in performance for *r* = 1, *r* = 2. We recommend using a rank of at least 4 when FT PLMs.

## Language Modeling in Biology.

While the first PLMs (Bepler & Berger, UniRep) used recurrent neural networks like the bi-LSTM (1, 16, 17), recent work has converged around masked language modeling and the transformer. Models like ProtBert, ProtT5 (18), and ESM (19) are transformers trained on massive sets of protein sequence data in an unsupervised manner. These models learn meaningful representations which can be applied to replace manual feature engineering, or computationally expensive evolutionary searches and construction of multiple sequence alignments. Most recently, ESM2 (3) represents the largest PLM to date, with models as large as 15 billion parameters. While this is still shy of the largest natural language models, this represents a significant step up in the size of PLMs and their capacity for unsupervised representation learning. While language modeling has seen the most success in proteomics, this success has seen language models expand to other aspects of biology. Biochemistry language models learn representations of small molecules (20, 21), most notably with ChemBERTa (22). Likewise, the release of models like scBERT (23), scGPT (24), and Geneformer (25) has led to advancements in single-cell genomics, and language models have also seen direct clinical use, such as with the medical question answering model Med-PaLM (26).

## Protein Structure.

One of the longest-standing challenges in computational biology is that of protein structure prediction. While proteins are modeled by language models as sequences of amino acids (primary structure), they fold into secondary (alpha helices, beta sheets) and more complex tertiary structures, which imbues them with a variety of functions. For nearly 30 y, the Critical Assessment of protein Structure Prediction (CASP) has measured the ability to computationally predict the tertiary structure of a protein from its primary sequence. In 2020, AlphaFold2 (27), closely followed by RoseTTAFold in 2021 (28), presented a massive jump in performance, reaching near-experimental levels of accuracy. AlphaFold2 and RoseTTAFold use multiple sequence alignments (MSA) to incorporate evolutionary context into structure prediction, and recent methods like OmegaFold (29) and ESMFold (3) instead use pretrained PLMs. While PLM-based approaches have yet to reach the accuracy level of MSA-based approaches across the board, they nonetheless achieve extremely accurate performance and require only a single sequence. Because of this, they are much more computationally efficient, forgoing the need for the expensive MSA search step. In addition, these methods often outperform MSA-based methods on intrinsically disordered regions or proteins with little evolutionary context and are competitive in terms of complex structure accuracy. Thus, PLMs represent an exciting step forward in tertiary structure modeling.

In addition to the structures that single chains fold into, proteins can form complexes known as quaternary structures comprising multiple protein chains. While methods like AlphaFold-Multimer (30) have attempted to fully model the structure of these complexes to moderate success, other methods have taken a different approach—focusing on the special case of homooligomeric proteins. In 2006, Levy et al. introduced 3DComplex (31), which categorizes protein complexes based on topology and symmetry. Recently, Schweke et al. introduced an atlas of protein homooligomerization (32), while QUEEN (33) attempts to predict the multiplicity of such complexes.

## Protein Interactions.

Cellular function is driven by a complex interplay of interactions between proteins. Experimental approaches to discern those interactions require substantial wet lab resources and time, which motivates the need for computational approaches to model protein interaction. Models such as AlphaFold-Multimer (30) have recently been developed to predict the structure of interacting complexes (34). Quaternary structure prediction is valuable if the pair is already known to interact, but often results in degenerate prediction for pairs which do not interact, and due to the size of the model is difficult to scale to the whole-genome and all possible protein pairs. Methods like PIPR (12), D-SCRIPT (13), Topsy-Turvy (35), and RAPPPID (14) predict PPI solely from widely available primary sequence and are fast enough to run at genome scale. Recent work has begun to close the gap between whole-genome interaction prediction and complex structure modeling (36, 37), potentially unifying genome-scale PPI prediction with complex structure prediction. The Human Reference Interactome (HuRI) (38) remains the most complete experimentally verified human protein interaction network.

## Results

### Reduced Memory Usage of PEFT Enables Deeper FT.

**Fig. 2.**
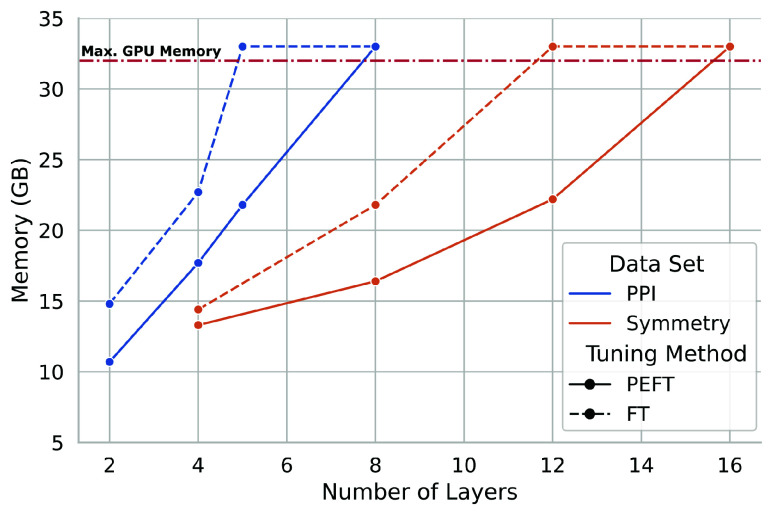
PEFT training requires reduced GPU memory. We compare the maximum GPU memory usage of PEFT vs. traditional FT different numbers of transformer layers. All values are reported in GB. Values above 32 GB (the red, dash-dot line) indicate that the run was killed due to running out of GPU memory. More layers are able to be adapted across the board for the symmetry prediction task since it requires only a single protein, rather than a pair. For the same number of layers, PEFT models require less GPU memory, thus PEFT enables adaptation of deeper model layers within the same limits of computational resources. We show in *SI Appendix*, Section S1.C that this deeper training often yields improved performance.

The most common approach to FT is to unfreeze the weights of the last *n* transformer layers, which we compare with adding LoRA weights to the last *n* layers. Fig. 2 shows the maximum GPU memory used by PEFT or traditional FT for the last *n* layers of an ESM2 model trained on PPI prediction or homooligomer symmetry prediction (see *SI Appendix*, section S2.B for further discussion of the FT approach). Theoretically, tuning only intermediate layers without adjusting weights that rely on those intermediate representations reduces the degrees of freedom available to the model, and we show this to be empirically true as well (*SI Appendix*, section S1.C). Both PEFT and FT eventually overflow available GPU memory as the number of layers tuned increases; tuning all layers of the model requires additional model parallelism with either approach. However, this parameter-efficient approach allows for training of deeper layers of the model while staying within a lower memory budget. Because the PPI task requires storing the compute graph for a pair of proteins, the marginal impact of PEFT methods on memory consumption is less than for the symmetry task, where using LoRA to adapt weight matrices allows for several additional layers to be trained. All experiments were performed on a GPU with 32 GB of memory, with a batch size of 4 and maximum sequence length of 1,024. In general, we show that adaptation of deeper layers results in a corresponding increase in performance. We specifically adapt increasingly many layers from the end of the model, rather than intermediate layers (*SI Appendix*, section S1.C).

### Parameter-Efficient Classifiers Achieve State-of-the-Art PPI Prediction.

We use PEFT to train an ESM2 model to predict PPIs from sequence on the benchmark dataset from Bernett et al. (15). We compare with a model that is fine-tuned in the traditional way (FT), and with a baseline that trains a classifier on sequence embeddings from the ESM2 model with frozen weights (MLP). See Fig. 1*A* for an overview of these three approaches. In addition, we compare to the best prior scores from Bernett et al. (15)—either Topsy-Turvy (35), or SVM-PCA, a baseline constructed by Bernett et al. (15) which trains a support vector machine on PCA-reduced sequence similarity vectors. Note that for each benchmark metric, we selected the best score across all methods evaluated, and that no single method achieved the “Best Prior” performance across the board, so this is a significantly higher threshold than comparing to any single method.

Table 2 shows the performance of these models. Both the PEFT and MLP models achieve performance competitive with the FT model, despite having several orders of magnitude fewer parameters. In fact, the MLP model achieves the best overall accuracy, MCC, and AUPR on these benchmarks, while the PEFT model achieves the best F1 score and recall. Surprisingly, both the MLP and PEFT models outperform the Best Prior methods in these metrics. These results suggest that parameter-efficient methods provide competitive alternatives and can unlock the predictive power of PLMs—and provide additional evidence that simple scaling of model size is not sufficient for proteomic tasks (39) (see also *SI Appendix*, section S1.A). Both PEFT and MLP models use less GPU memory than the FT model (MLP substantially so) and the MLP model requires significantly less training time. The reduced GPU memory usage of the PEFT model allows us to train deeper layers of the model (Fig. 2 and *SI Appendix*, section S1.C).

### Predicting Homooligomer Symmetry with PEFT.

We additionally train PEFT, FT, and MLP models to predict homooligomer symmetry. While this task also involves learning representations that capture protein structure, it is fundamentally different from the PPI task because it requires learning on only a single protein, rather than a pair. As we show in Table 1, both the PEFT and FT models significantly outperform the MLP model on all metrics. Traditional FT still yields the best performance, but PEFT using LoRA is a viable alternative—performance is within 10 to 15% of the FT model, and the model uses three orders of magnitude fewer parameters. The PEFT model actually achieves the best MCC and specificity of the three. Because homo-oligomer symmetry prediction is a less-well-studied problem, there is not a clear set of best prior methods with which to compare—in concurrent work, we introduce a computational method Seq2Symm (40) that is specifically optimized for this problem.

**Fig. 3.**
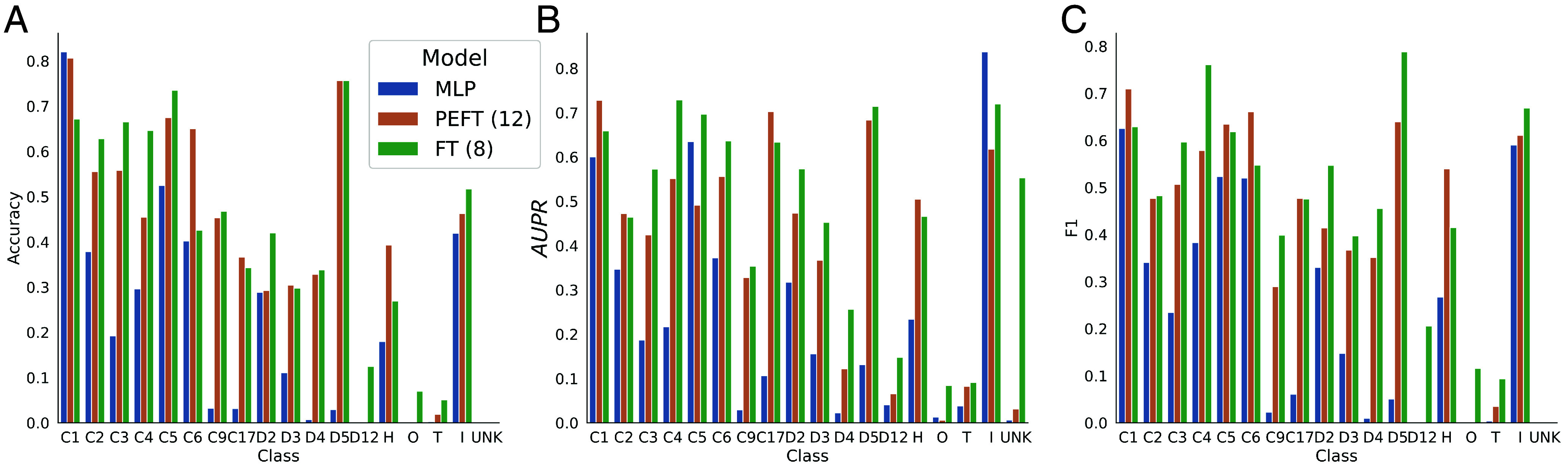
Breakdown of model performance by homooligomer symmetry class. Looking at only a single set of metrics can be helpful to get an aggregate idea of model performance, but hides complexity in the case of a multiclass classification task such as homooligomer symmetry prediction. Here, we show the per-class accuracy (*A*), AUPR (*B*), and F1 (*C*) of a classifier trained with frozen ESM2 embeddings (MLP), a model fine-tuned with LoRA (PEFT, 12 layers), and a model where all parameters in the final 8 layers were fine-tuned (FT). MLP performance is unsurprisingly relatively strong for high-support “easy” classes (e.g., C1, C5, D2, I), but substantially worse for rarer classes (e.g., C7–9, C10–C17, D4, D5). The PEFT model is competitive for most classes, but generally lags behind the performance of the FT model. We report the support of each class, which roughly corresponds to the AUPR of a random classifier, in *SI Appendix*, Table S4.

Unlike the binary prediction task of PPI prediction, homooligomer symmetry prediction is a multiclass problem, where we consider 17 different possible symmetries (and an 18th “Unknown” class). This makes it more difficult to summarize model performance into a single number—especially because the number of examples of different classes in the test set is so variable (*SI Appendix*, Table S4). The numbers reported in Table 1 are macroaverages across all classes, but in Fig. 3, we show the accuracy, AUPR, and F1 score of the three models broken down by true class. These results make it clear that the improved performance of the PEFT and FT models relative to the MLP baseline comes primarily from their increased ability to learn about rare classes. On common classes like C1, C5, D2, and I, the MLP model achieves accuracy competitive with the PEFT model and often even the fine-tuned model. However, the larger complexity of the FT model allows it to also learn about classes like C3, C4, D6–D12, and O significantly better than the less complex models. This may partially explain why performance between the three models is much closer in the balanced PPI prediction task.

### Visualizing Attentions After FT.

**Fig. 4.**
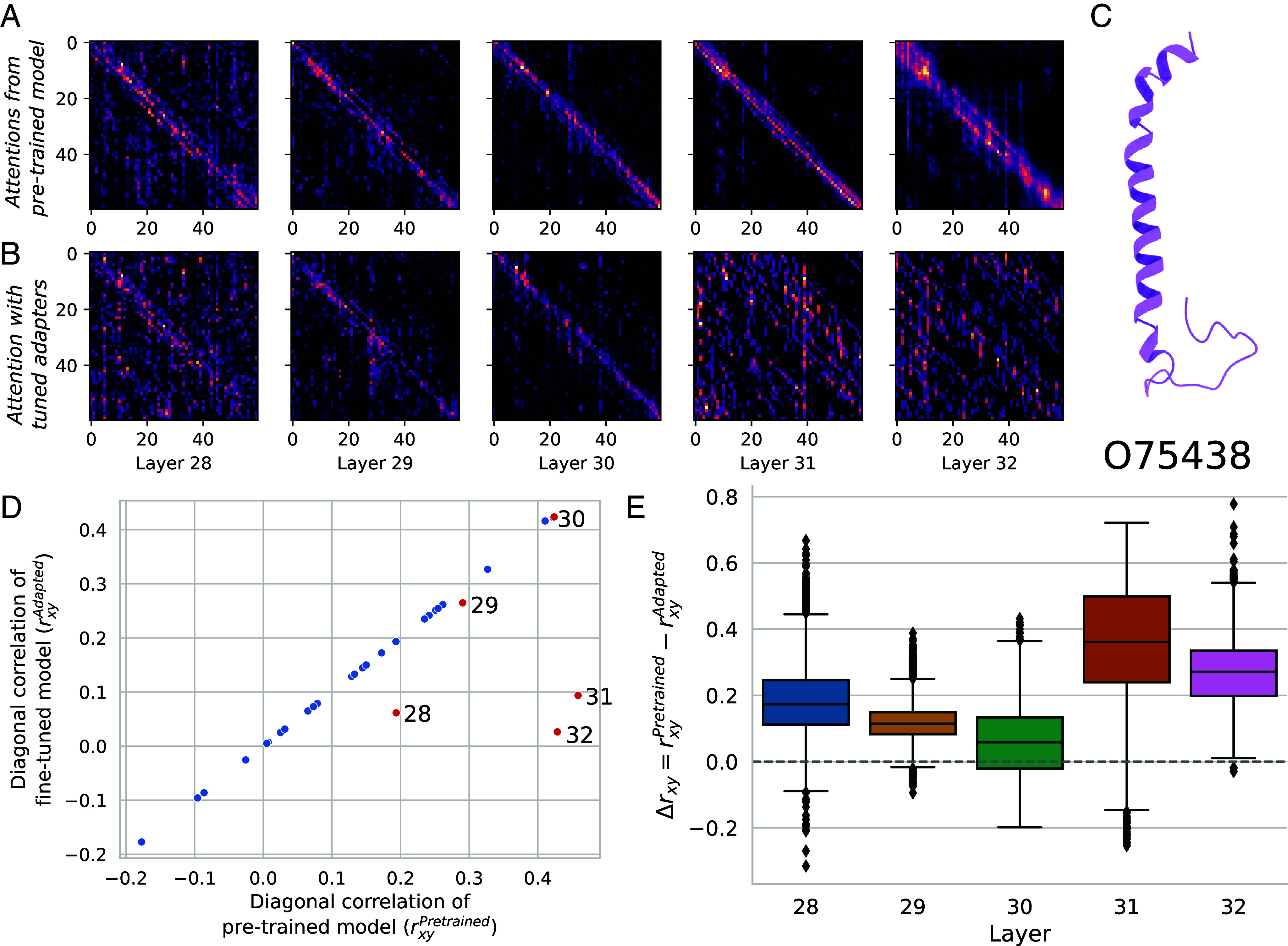
Visualizing attention matrices. (*A*) Attentions for NADH dehydrogenase 1 *β* subcomplex subunit 1 (UniProt ID: O75438) using the pretrained ESM2. (*B*) Attentions for the same protein after PEFT. (*C*) Structure of O75438. We find that PEFT weights result in attention that is more spread out across the length of the protein when trained for PPI prediction. (*D*) For the attention values in each of the 32 layers, we compute the extent to which attention is concentrated along the diagonal (local) as opposed to diffused (global) using the sample Pearson correlation (*r*_*xy*_). For the final five layers which have been adapted with PEFT, this diagonal correlation is significantly lower indicating more globally diffuse attention. (*E*) We compute the difference between the diagonal correlation of the base and adapted attentions (Δ*r*_*xy*_) for all 3,020 proteins in our PPI dataset. We find that attentions in every layer are significantly less diagonal after FT, with the strongest impact in the final two layers.

In Fig. 4, we show the impact of PEFT on attention (*A* matrix from Fig. 1*D*). Here, we look at the PEFT model trained for PPI prediction from Table 2. We visualize attention from the five LoRA-adapted layers averaged across all heads. Fig. 4*A* shows attention from the pretrained model for NADH dehydrogenase 1 *β* subcomplex subunit 1 (UniProt ID: O75438) (Fig. 4*C*). Attention is concentrated along the diagonal. In contrast, after PEFT with LoRA weights the attention is much more diffuse across the length of the protein, especially in the later transformer layers (Fig. 4*B*). This suggests that for PPI prediction, the LoRA weights allow for more distant amino acids to attend to one another. We show one representative example here, but this diffusion of attention occurs broadly; we show another representative example (NADH dehydrogenase 1 *β* subcomplex subunit 10, UniProt ID: O96000, which interacts with O075438 in our test set) in *SI Appendix*, Fig. S5.

We can quantify this effect by computing the sample Pearson correlation of these attention values. In summary, this value treats attention values at coordinates *i, j* as samples from distributions *X, Y* and computes the correlation of these samples (see *SI Appendix*, section S3 for details). This value measures the extent to which attention is concentration near the diagonal, capturing local vs. global effects of attention. We show a decrease in correlation along the diagonal after FT (Fig. 4*D*) for the five layers which are adapted using LoRA. We then compute this correlation for the pretrained PLM and the adapted PLM for all 3,020 proteins. Fig. 4*E* shows the distribution of differences in scores for each layer, where a higher value indicates more global attention. For all layers adapted, there is a significant difference in the diagonal concentration of attention after the application of PEFT. This suggests that the representations produced by the language model are able to predict PPI in part by increasing the relative impact of long-range attention. This effect is similar to what is seen when FT all parameters of the PLM (*SI Appendix*, Fig. S6). For symmetry, this effect is less pronounced, perhaps because homooligomer assembly is often guided primarily by local rules (41) (*SI Appendix*, Fig. S7).

### Impact of LoRA Hyperparameters on Performance.

In their original manuscript on LoRA, Hu et al. (9) show that for natural language models, adding low-rank adapter matrices to only the query and value weights (*W*_*Q*_, *W*_*V*_) of the attention heads yields the best tradeoff of performance and parameter-efficiency. However, the space of natural language is not necessarily the same as that of protein sequence, and we sought to evaluate to what extent the choice in adapted weight matrices affects performance. Table 4 shows that while performance is relatively robust to the choice of weight matrices, adapting the key (*W*_*K*_) and value (*W*_*V*_) matrices results in the best overall performance. We note that the value matrix alone achieves similar results while also being more parameter-efficient. Thus, that could be the best choice for PEFT of PLMs, if memory constraints are especially tight. When applying LoRA to PLMs, we recommend adding adapter matrices to the key and value weight matrices.

**Table 4.** Adaptation of different sets of transformer weights

Weight matrix	# Trainable params.	Val. AUPR	AUPR	Acc.	F1	MCC	Prec.	Rec.	Spec.
*W*_*Q*_	266,597	0.536	0.529	0.516	0.430	0.034	0.524	0.364	0.669
*W*_*K*_	266,597	0.565	0.562	0.544	0.433	0.095	0.572	0.348	**0.739**
*W*_*V*_	266,597	0.612	0.610	**0.605**	**0.650**	**0.216**	0.583	**0.735**	0.474
*W*_*Q*_, *W*_*K*_	368,897	0.590	0.576	0.564	0.582	0.129	0.559	0.607	0.521
*W*_*Q*_, *W*_*V*_	368,897	0.619	0.617	0.599	0.633	0.201	0.583	0.692	0.506
*W*_*K*_, *W*_*V*_	368,897	**0.637**	**0.633**	0.601	0.630	0.205	**0.587**	0.680	0.522
*W*_*Q*_, *W*_*K*_, *W*_*V*_	471,297	0.628	0.613	0.603	0.639	0.210	0.585	0.704	0.502

Hu et al. (9) recommend adding low-rank adapters to the query and value matrices of the attention heads for natural language. We investigate whether the same recommendation holds for the space of protein language, specifically on the task of PPI prediction. We report the validation AUPR, which was used to select the best epoch for test set evaluation, and the AUPR, accuracy, F1 score, MCC, precision, recall, and specificity on the test set for all different combinations of the query (*W*_*Q*_), key (*W*_*K*_), and value (*W*_*V*_) matrices. We find that adapting the *W*_*K*_ and *W*_*V*_ together yields the best performance. Adapting only the value matrix also performs well and uses fewer parameters.

The rank *r* of the newly added LoRA weight matrices plays an important role in the performance of PEFT models and the memory that they require. Hu et al. (9) show that LoRA remains effective at extremely low ranks, with competitive performance even when *r* = 1. However, the effectiveness of different rank values is dependent on both the intrinsic dimension of the language and the task (42), and robustness in rank variance will not necessarily hold for proteomic sequences and inference tasks. In Table 3, we show the results of training PEFT models with LoRA rank *r* = 1, 2, 4, 8, 64 [following Hu et al. (9)] to predict PPIs. We find that the best model performance is achieved at *r* = 4, but that higher ranks yield similar performance. For ranks less than 4, model performance is still strong, but is noticeably reduced. We perform the same experiments on homooligomer symmetry prediction, with similar findings—although *r* = 8 is better for this task (*SI Appendix*, Table S5).

## Discussion

In this work, we bring PEFT methods to PLMs. We show that PEFT models achieve performance comparable to traditional FT, while requiring a reduced memory footprint, which enables training deeper layers of the model. The competitive performance of PEFT models holds both for PPI prediction, a balanced, binary task, as well as symmetry prediction, a multiclass and highly unbalanced task. Our work democratizes PLMs by charting a path for FT these models using substantially fewer parameters and less GPU memory. As the scale of PLMs continues to increase, it will become increasingly important to use these and other approaches when traditional FT is computationally infeasible.

Additionally, while approaches from natural language processing have so far transferred quite well, the distributions and intrinsic dimensions of protein language are clearly different than natural language. We show one consequence of this; our recommendation for LoRA rank, *α*, and which attention matrices to adapt differs from the original conclusions of Hu et al. (9). Notably, LoRA when applied to proteomics does not seem to admit as low of a rank as suggested in the initial report. In considering the question of which matrices to adapt, while we see consistent results between the two tasks we test, it is possible that these conclusions will vary with additional tasks. While our recommended parameters offer a strong starting point, it remains important to test these parameters with a validation set to achieve maximum performance when adapting large foundation models. Further, while LoRA is currently the most widely used PEFT method for natural language, other PEFT methods exist which may better suit the space of protein language such as the adapter method of Houlsby et al. (10). QLoRA (43) is an especially promising approach that performs model quantization to substantially reduce memory usage. This quantization will likely be especially valuable for natively memory-intensive tasks, such as modeling extremely long proteins or prediction of protein complexes. It may be necessary in the future to develop parameter-efficient approaches specific to protein language.

The successful transfer for PEFT from large language models to PLMs suggests that other efficiency techniques such as quantization may yield similar performance gains (44, 45). While this work has focused on adaptation of PLMs, large protein structure models such as OmegaFold, RosettaFold, ESMFold, and AlphaFold (3, 27–29) have begun to see use as foundation models and could be similarly amenable to tuning for downstream tasks with PEFT methods. In addition, it remains to be shown whether PEFT methods are equally competitive for language models trained on other types of biological sequences, such as DNA (46) or SMILES strings (22).

This work also shows the limitations of scale in PLM. Not only are PEFT and MLP models able to achieve state-of-the-art performance on PPI prediction, but we show that embeddings from the 650 million parameter version of ESM2 outperform those from the 3 billion parameter version (*SI Appendix*, section S1.A). While larger models certainly enable better performance, they do not guarantee it; we emphasize the continued need for models which can be easily trained and run by even small research groups, including primarily biological groups with constrained computational resources. These limitations of scaling laws for PLMs also suggest that data, rather than compute, is presently the bottleneck in performance. The training datasets used for PLMs are still orders of magnitude smaller than the largest natural language training datasets. Thus, an increase in the number and diversity of protein sequences, such as from metagenomes (3, 47), could drive improved performance for larger PLMs. The inference-time efficiency of the MLP method, which only requires a single forward pass through the language model per protein, is especially valuable for tasks which require ultramassive prediction, such as large compound library screens (48, 49). Especially with the cost and environmental impact of large-scale language model training and tuning (50), parameter-efficient tuning or methods which use frozen embeddings should remain a viable alternative for tailoring foundation models to a specific task, and should be tested alongside traditional FT approaches.

## Materials and Methods

### Benchmark Data: PPI.

While creating train/test splits based on filtering homologous proteins is common in machine learning for proteomics, the binary nature of PPI prediction presents a unique challenge because data leakage can still occur if only one protein of an interacting pair appears in both sets. If a so-called “hub” protein with many interactions appears in both the training and test set, models can learn that this specific protein is likely to have positive interactions. Then, test set performance will be inflated even if nothing is learned about the actual pairwise interactions. After noting pervasive biases in previous benchmarks relating to sequence similarity and node degree, Bernett et al. (15) introduced a new gold standard dataset for benchmarking PPI. The splits introduced in this benchmark apply a more stringent notion of sequence similarity for pairwise problems as introduced by Park and Marcotte (51), splitting by C3 similarity. In addition, both the positive and negative datasets are balanced with regard to node degree; as a consequence models cannot learn that proteins *in general* interact just because they are high degree. This dataset consists of 163,192/59,246/52,035 training/validation/test edges, with an 1:1 ratio of positives to negatives.

### Benchmark Data: Homooligomer Symmetry.

The multiplicity and symmetry prediction task is formulated as a multiclass prediction problem. Given a protein chain, we classify it as one of 17 symmetry classes C1,C2,C3,C4,C5,C6,C7−C9,C10−C17,D2,D3,D4,D5,D6−D12,H,O,T,I (or “Unknown”). The *C* classes correspond to cyclic symmetries, the *D* classes to dihedral symmetries, and H, O, T, and I to helical, octahedral, tetrahedral, and icosahedral symmetries, respectively. More extensive detail on different symmetry classes can be found within 3DComplex (31). Protein sequences and structures were obtained from the Protein Data Bank (PDB) (52), as were their labels. Sequences were clustered at 30% sequence similarity and 80% coverage using MMseqs2 (53), and these clusters were used to define train, validation, and test splits. This method of splitting ensures that no two sequences that are highly similar will be in both a training and evaluation set, which would allow the model to memorize sequence similarity rather than learning properties of sequence and structure that correspond with symmetry. These data consist of 370,986/46,833/102,978 training/validation/test edges. The support for each class in the text set is described in *SI Appendix*, Table S4.

We tracked performance of models through multiple metrics. It is difficult to pin performance to a single number with a multiclass classification, especially when the support for different classes is highly imbalanced. We compute the accuracy, F1 score, MCC, average precision (AUPR), precision, recall, and specificity for each class, as well as metrics averaged over each class. Note that because class support is highly variable (*SI Appendix*, section S2.A) and resulting model performance varies widely, we use an unweighted (“macro”) average to capture performance broadly across all classes.

### PLM.

We focused our efforts on ESM2, a transformer-based PLM which is presently considered the state-of-the-art in PLM (3). ESM2 has several different model sizes, ranging from eight million to 15 billion parameters. For this study, we focused on the 650 million parameter version of ESM2 (*SI Appendix*, section S1.A).

For an amino acid sequence $X=x_{1}x_{2}...x_{n}$, a PLM of dimension *d* returns a set of embeddings $E\in R^{d\times n}=e_{1}e_{2}...e_{n},e_{i}\in R^{d}$. To standardize the size of representations for sequences of dynamic length, a pooling step needs to be undertaken. This is most commonly done either by averaging along the length of the sequences ($e_{p}\in R^{d}=\frac{1}{n}\sum_{i=1}^{n}e_{i}$) or by selecting the first token of the sequence, a non–amino acid token ([clsf]) created specifically for sequence classification. Here, we chose to take the former approach as it explicitly integrates signal across the length of the protein. We note that while this is a commonly used approach, how to best aggregate sequence-length representations into a fixed dimension embedding is an open problem in language modeling [see Naderi Alizadeh and Singh (54) for one recently proposed approach]. Converting this pooled embedding into a binary ($Y\in R^{2}$) or multiclass ($Y\in R^{18}$, 17 possible symmetry classes + “Unknown”) prediction requires an additional classification head. For the PPI prediction task, fixed-length embeddings were averaged before being passed to the classification head, as in Szymborski et al. (14). For the symmetry prediction task, there is only a single protein, so embeddings were passed directly to the classification head. In this study, we tested two different prediction heads. The first, applied directly to the pretrained *E* without FT, is a simple MLP, with the number and size of layers determined by grid search (*Materials and Methods* and *SI Appendix*, section S1.E). The second is the ESMClassificationHead made available by the authors in the public HuggingFace repository, which consists of two dense layers with dropout and a tanh activation between the layers. We selected classification heads which were demonstrated to yield strong performance in previous work in order to minimize the need for hyperparameter search in this space.

### Parameter-Efficient Adaptation.

Houlsby et al. (10) introduced adapters, which add parameters in serial to each transformer layer, allowing for every layer of the model to be trained using only a small number of parameters. The current state-of-the-art is low-rank adapters (LoRA), introduced by Hu et al. (9), which adds two low-rank adapter matrices in parallel to the query and value weight matrices of the attention heads (Fig. 1*A*, *Middle*). LoRA adds two low-rank matrices *A* and *B* to each adapted weight matrix. Given weight matrix $W\in R^{d\times k}$, LoRA adds new parameters $A\in R^{r\times k},B\in R^{d\times r},r\ll d,k$. The normal forward pass of the layer given input $x\in R^{k}$ is h=Wx, and the forward pass with the LoRA adaptation is h=Wx+BAx. Only the weights of A,B are updated during backpropagation, while the weights of *W* are frozen. BAx is scaled by the quantity $\frac{\alpha }{r}$, where *α* is a hyperparameter which is held constant in the original report. While *A* is initialized with a random Gaussian distribution, by initializing B=0 the first forward pass of the model is equivalent to the pretrained model without adaptation. Following the recommendations of the original paper, we initially apply LoRA only to the query and value matrices of the attention head. We explore different combinations of weight matrix adaptation and different values of the rank *r*. Even though *α* is originally held constant, we evaluate different settings of the *α* parameter as they relate to different rank values in *SI Appendix*, section S2.D.

### Training and Implementation.

All PEFT and FT models were implemented in PyTorch (v.2.0.1), using the HuggingFace implementations of ESM2 from the transformers package (v.4.32.1) and LoRA from the peft package (v.0.5.0). Models were primarily trained on NVIDIA V100 GPUs with 32GB of and occasionally on NVIDIA A100 GPUs with 80GB memory, although all memory-usage data presented here is from V100s. We used the binary cross-entropy with logits loss to compute error, with an L2 weight decay of 0.01. Model weights were optimized via backpropagation using the Adam optimizer and a cosine decay with restarts learning rate schedule (initial learning rate 0.001 for MLP and PEFT, 0.0005 for FT). Models were trained with an epoch size of 16,384, with an on-device batch size of 4 and gradient accumulation every 16 steps, for an effective batch size of 64. PEFT and FT models were trained for 40 epochs and the best model based on validation AUPR was chosen for testing. Except for where otherwise specified, we used LoRA r=8, LoRA α=32, LoRA dropout P=0.1.

The hyperparameters for the MLP on language model embeddings with frozen weights were chosen by a grid search [implemented in scikit-learn (v.1.2.0), *SI Appendix*, section S1.E]. The best-performing model was constructed with two hidden layers with sizes (64,64) and ReLU activations and was optimized with the Adam optimizer for 2,000 iterations with a tolerance of 0.0001 and an adaptive learning rate initialized at 0.01.

## Supplementary Material

Appendix 01 (PDF)

## Data Availability

All model adaptation, training, and benchmarking code is made available open-source on GitHub at https://github.com/microsoft/peft_proteomics. Previously published data were used for this work (15, 52).
