# Supplementary material for: Democratizing protein language models with parameter-efficient fine-tuning
Source: Proc Natl Acad Sci U S A. 2024 Jun 20;121(26):e2405840121. doi: 10.1073/pnas.2405840121 (PMC11214071; doi:10.1073/pnas.2405840121)
Supplement: Supplementary file 1 — Appendix 01 (PDF) [file pnas.2405840121.sapp.pdf]

Supporting Information for

Democratizing Protein Language Models with Parameter-Efficient Fine-Tuning

Samuel Sledzieski, Meghana Kshirsagar, Minkyung Baek, Rahul Dodhia, Juan Lavista Ferres, Bonnie Berger

Juan Lavista Ferres, Bonnie Berger.
E-mail: jlavista@microsoft.com, bab@mit.edu

This PDF file includes:

Supporting text
Figs. S1 to S7
Tables S1 to S6
SI References

Supporting Information Text

S1. Protein-Protein Interaction Prediction

A. 650M vs. 3B ESM2 Model. Typically in language modeling, larger models yield better performance leading to training or increasingly large models. We trained a multi-layer perceptron classifier (MLP) on embeddings from both 650M and 3B parameter models with frozen weights to predict protein-protein interactions. We found that despite having 4.5x fewer parameters, the 650M parameter model actually performs slightly better (Table S1). This indicates that even with reduced compute capacity available, smaller foundation models may be sufficient to achieve good performance on proteomics tasks—and that the limiting factor for performance may not simply be the scale of models. Consequently, all results presented elsewhere in this manuscript use the 650M parameter version of ESM2. We discuss the question of foundation model size in the Discussion.

Table S1. Test set performance of a MLP classifier trained on pooled embeddings from the 650 million and 3 billion parameter versions of ESM2 with frozen weights. While the two models are competitive, the 650M parameter version outperforms the larger 3B parameter version in accuracy, MCC, AUPR, precision, and specificity. The 3B parameter model achieves a higher F1 score and recall.

	Accuracy	F1	MCC	AUPR	Prec.	Rec.	Spec.
**650M**	**0.631**	0.632	**0.261**	**0.684**	**0.630**	0.633	**0.623**
**3B**	0.607	**0.650**	0.221	0.656	0.586	**0.730**	0.484

B. Learning rate selection for fine-tuning. Due to the substantially larger number of parameters, fine-tuned models are more prone to over-fit to the training data. As such, it is common to use lower learning rates when performing full fine-tuning compared to training only the classification head (MLP) or a small number of added paramters (PEFT). In Table S2, we show the result of fine-tuning with several different learning rates. The results presented in the main text (Table 1) uses a learning rate of $5e-4$.

Table S2. Fine-tuning a larger number of parameters typically requires a lower learning rate to reduce over-fitting. We experiment with 5 different learning rates from $1e-3$ to $1e-5$, finding the best performance at a learning rate of $5e-4$, half of what is used for MLP and PEFT models.

Learning Rate	Val. AUPR	AUPR	Acc.	F1	MCC	Prec.	Rec.	Spec.
1e-3	0.605	0.576	0.548	0.568	0.097	0.544	0.594	0.502
5e-4	**0.622**	**0.623**	**0.604**	0.631	**0.210**	0.591	**0.676**	0.532
1e-4	0.615	0.604	0.603	0.622	0.207	**0.594**	0.653	0.553
5e-5	0.619	0.596	0.562	0.480	0.130	0.590	0.405	**0.718**
1e-5	0.532	0.536	0.500	**0.667**	0.000	0.500	1.000	0.000

C. Deeper layer adaptation correlates with increased performance. One choice when selecting designing a parameter-efficient fine-tuning setup for a protein language model is which and how many transformer layers to adapt. We show in Table S3 that, on the PPI prediction task, increasing numbers of adapted layers (with more parameters) leads to generally stronger performance (although this is not a uniform increase due to variance in model training). However, it is not immediately clear that the best strategy is to adapt subsequent layers from the end. Although this is theoretically sound, we compare the following alternative approaches on the symmetry prediction task (test set AUPR in parenthesis): adapting the last 5 layers (0.400), adapting the first 5 layers (0.215), adapting 5 intermediate layers (layers 14-19; 0.344), adapting 5 randomly selected layers (layers 3, 6, 12, 17, 23; 0.332). We find significantly degraded performance with all options except for the last 5 layers. Performance is especially bad when adapting only the first 5 layers. This corresponds with the generally-understood principle that early layers of a protein language model learn broad abstractions of the data, while later layers learn task-specific features.

D. PEFT and FT Training and Validation Curves. We show training and validation loss curves, as well as validation AUPR curves, over training in Figure S1. In Figure S2, we show training and validation loss curves, as well as validation AUPR curves, for all different combinations of Q/K/V matrices tested in Table 3. In Figure S3, we show training and validation loss curves, as well as validation AUPR curves, for LoRA rank $1, 2, 4, 8, 64$ as tested in Table 4.

Table S3. We find that model performance generally increases as increasingly many layers are adapted from the end of the model.

# Layers	Val. AUPR	AUPR	Acc.	F1	MCC	Prec.	Rec.	Spec.
1	0.612	0.605	0.524	0.586	0.171	0.586	0.586	**0.586**
2	0.615	0.622	0.602	0.632	0.208	0.589	0.681	0.524
4	0.619	0.597	0.588	0.630	0.180	0.572	0.701	0.475
5	**0.640**	**0.633**	0.601	0.630	0.204	0.587	0.680	0.522
6	0.627	0.624	**0.604**	**0.643**	**0.213**	0.586	**0.711**	0.497

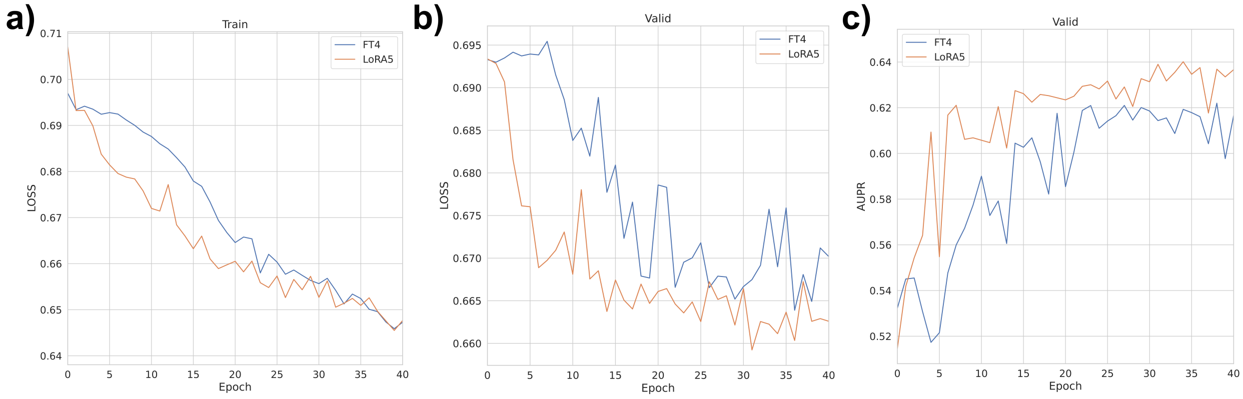

Fig. S1. Training **(a)** and validation **(b)** loss curves, AUPR curves **(c)** for FT (4 layers) and PEFT (5 layers) from Table 1. Note that all other training parameters were held constant between these runs.

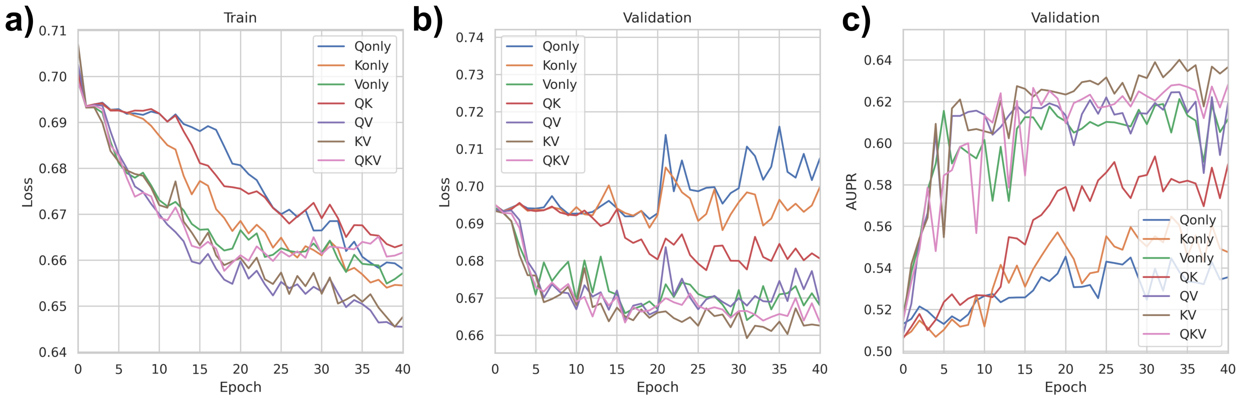

Fig. S2. Training **(a)** and validation **(b)** loss curves, AUPR **(c)** curves for models trained with LoRA adapters on Q, K, V, QK, QV, KV, QKV matrices from Table 3. Note that all other training parameters were held constant between these runs.

E. Baseline MLP Model. As a baseline to compare with fine-tuning, we train an MLPClassifier model from scikit-learn using embeddings extracted from ESM2 (650M parameters). Parameters for the MLPClassifier were selected by cross-validation on macro average precision over a grid search. We searched over all combinations of

- $activation = [“logistic'', “relu'', “identity'']$
- $alpha = [0.0001, 0.001, 0.01]$
- $learning\_rate\_init = [0.001, 0.01]$
- $max\_iter = 1000, 2000$

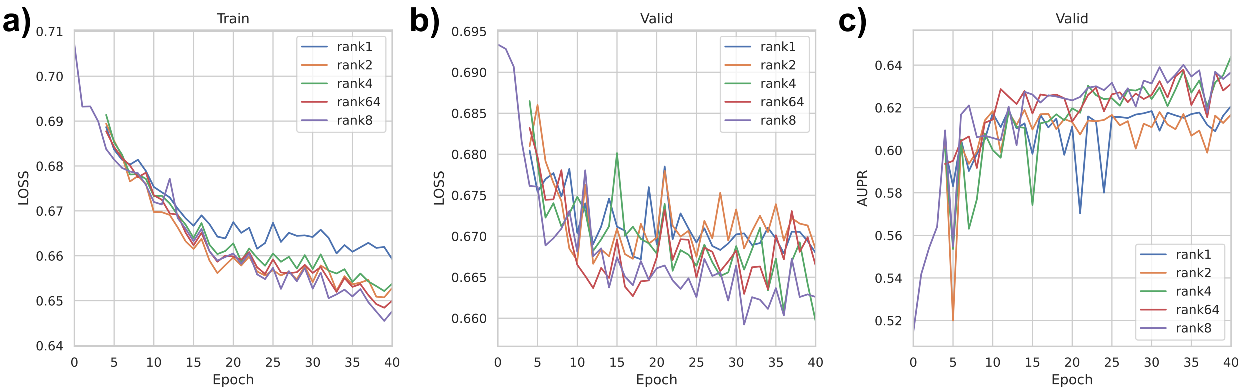

Fig. S3. Training **(a)** and validation **(b)** loss curves, AUPR **(c)** curves for models trained with LoRA ranks $r = 1, 2, 4, 8, 64$ from Table 4. Note that all other training parameters were held constant between these runs.

- $hidden\_layer\_sizes = [(64,), (128,), (512,), (64, 64), (128, 128), (64, 64, 64)]$
- $tol = [1e-4, 1e-5]$

In Figure S4, we show that this MLP model is well calibrated (fraction of predicted positives at threshold p roughly matches predicted probability p).

S2. Homooligomer Symmetry Prediction

A. Test Set Support. Homooligomer symmetry prediction is a highly unbalanced, multi-class prediction task. In Table S4, we show the number of examples in the test set for each class. We show in Figure 3 that PEFT and MLP models are competitive for high support classes like C1, C2, C5, D2, and I, while the FT model is substantially better on rare classes like C7-C9, D4, D5, and O.

Table S4. Test Set Support. Number of examples of each symmetry class in the test set. This data is highly imbalanced, with most examples having either C1, C2, D2, or D3 symmetry.

Symmetry Class	Support	Random Classifier AUPR
C1	28,899	
C2	20,671	
C3	4,666	
C4	3,057	
C5	5,955	
C6	2,885	
C7-C9	1,406	
C10-C17	1,910	
D2	9,384	
D3	7,539	
D4	1,377	
D5	1,700	
D6-D12	1,975	
H	3,954	
O	520	
T	1,895	
I	4,857	
Other	328	

B. Fine-tuning all layers. We perform an additional experiment wherein we fine-tune *all* layers of the protein language model– we note that this is substantially more compute intensive and requires us to decrease our batch size to 4. We also reduce the learning rate to 0.0005 to account for the larger number of parameters. We find that on the symmetry

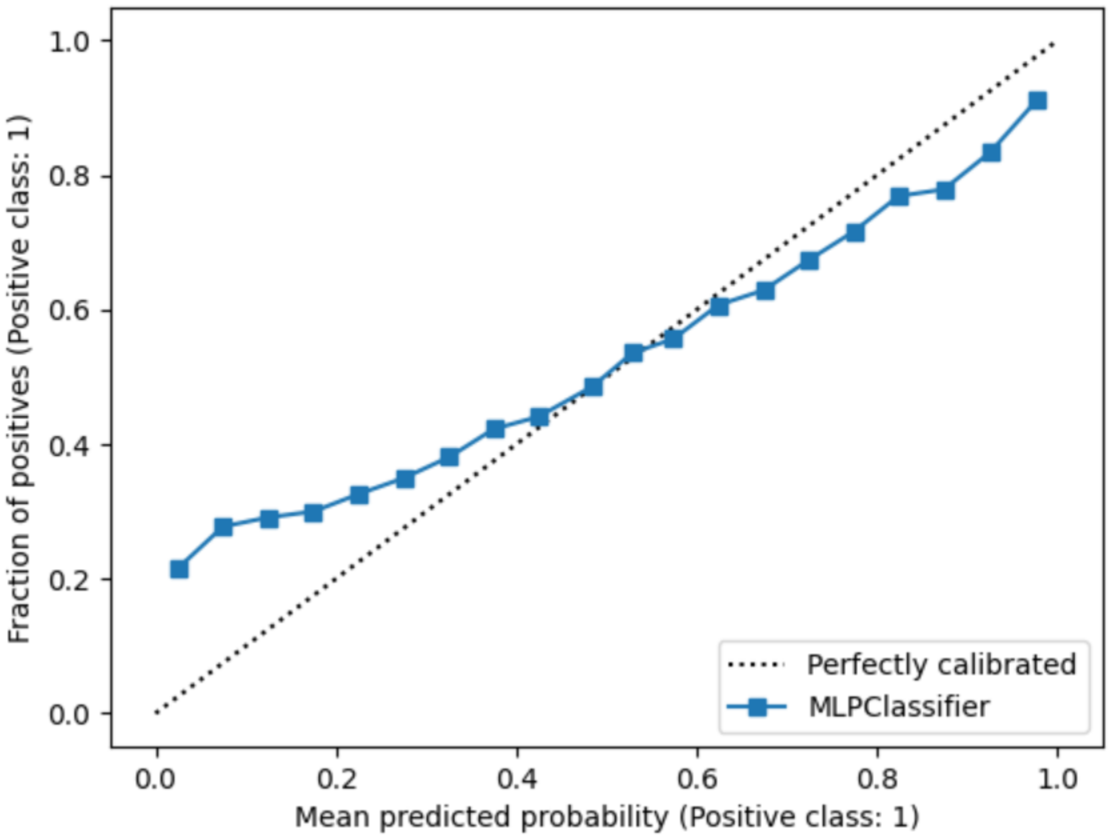

Fig. S4. Calibration curve for sklearn MLP on embeddings from a frozen ESM2 model, trained for PPI prediction on the benchmarks from Bernett et al. (1).

task, this model achieves a test set AUPR of 0.446, which is competitive with but does not exceed the performance of the models in Table 2. Thus, when we refer to "fine-tuning", we consider training all parameters of a fixed subset of layers in the model.

C. Symmetry Rank Experiments. We show in Table S5 the result of training models with different LoRA rank values for homooligomer symmetry prediction. Here, $r = 8$ is the best performing, closely followed by $r = 4$. Performance drops off noticeably with $r < 4$.

Table S5. Robustness in rank also holds for homooligomer symmetry prediction. We perform the same hyperparameter search over rank as we have previously done for PPI, this time training models to predict homooligomer symmetry. We find that like for PPI prediction, while performance is respectable at all values, it drops off noticeably for $r < 4$. For symmetry, rank $r = 8$ is the best performing, and there is actually a slight drop off with rank $r = 64$.

Rank	Val. AUPR	AUPR	Acc.	F1	MCC	Prec.	Rec.	Spec.
1	0.506	0.359	0.345	0.352	0.428	0.419	0.345	0.968
2	0.525	0.385	0.351	0.369	0.450	0.516	0.351	0.969
4	**0.531**	0.403	0.372	0.383	0.455	0.503	0.372	0.969
8	0.461	**0.416**	**0.390**	**0.430**	**0.468**	**0.558**	**0.390**	**0.970**
64	0.445	0.388	0.359	0.358	0.420	0.444	0.359	0.968

D. Impact of varying alpha at different ranks. In their original report, Hu et al. (2) state that the hyperparameter α functions similarly to a learning rate, and that they set it to the first rank value tried and hold it constant. However, we sought to evaluate whether tuning this parameter α could have an impact on performance. In Table S6, we show various combinations of α and rank applied to the symmetry prediction task.

Table S6. We report here the test set AUPR of several model trained with PEFT on the symmetry prediction task, with varying values of the α and rank hyperparameters of LoRA. While performance does vary with α and rank, there is not a clear correlation between the two.

α\Rank	1	2	4	8	64
**16**	0.395	0.404	0.394	0.399	0.377
**32**	0.395	0.385	0.403	0.416	0.387
**64**	0.414	0.429	0.420	0.423	0.430
**100**	0.376	**0.436**	0.422	0.431	0.418

S3. Visualizing Attention

We visualize attention values with and without parameter-efficient adapter updates for the last five transformer layers of the PEFT model trained on PPI prediction from Table 1. We average the output of all 20 attention heads so that for a protein of length n, we get a matrix of size $n \times n$. LoRA weights are turned on or off with the `peft` package commands `disable_adapter_layers` and `enable_adapter_layers`. We find that with fine-tuning on PPI prediction, attentions are spread out much further from the diagonal, indicating more distal attention necessary for predicting protein-protein interactions. We show representative examples in Figure 4 and Figure S5, a pair of interaction proteins from the NADH dehydrogenase 1 β subcomplex.

We compute a measurement of the extent to which attention is concentrated along the diagonal using the Pearson's sample correlation (3). Given a protein of length N, we treat the attention from residue i to residue j in matrix $A \in \mathbb{R}^{N \times N}$ as a pair of samples (i, j) from discrete distributions X and Y, where the magnitude of the attention $A_{i,j}$ corresponds to the probability mass of this pair of indices, or the weight of each sample. Then, the total "sample size" is the total magnitude of attention $n = \sum_{i,j \in N} A_{ij}$. We can then calculate the diagonal correlation of A as

$$r_{xy} = \frac{(n \sum_{i,j \in N} A_{ij} ij) - (\sum_{i,j \in N} A_{ij} i - \sum_{i,j \in N} A_{ij} j)}{\sqrt{n \sum_{i,j \in N} A_{ij} i^2 - (\sum_{i,j \in N} A_{ij} i)^2} - \sqrt{n \sum_{i,j \in N} A_{ij} j^2 - (\sum_{i,j \in N} A_{ij} j)^2}} \quad [1]$$

This effect is less noticeable in PEFT models trained to predict homooligomer symmetry, where there is a slight diffusion of attention but it still remains largely concentrated along the main diagonal (Figure S7).

References

1. Bernett J, Blumenthal DB, List M (2024) Cracking the black box of deep sequence-based protein–protein interaction prediction. *Briefings in Bioinformatics* 25(2):bbae076.
2. Hu EJ, et al. (2021) LoRA: Low-rank adaptation of large language models. *arXiv preprint arXiv:2106.09685*.
3. (https://math.stackexchange.com/users/85024/tad) T (year?) Measure of how much diagonal a matrix is (Mathematics Stack Exchange). URL:https://math.stackexchange.com/q/1393907 (version: 2015-08-12).

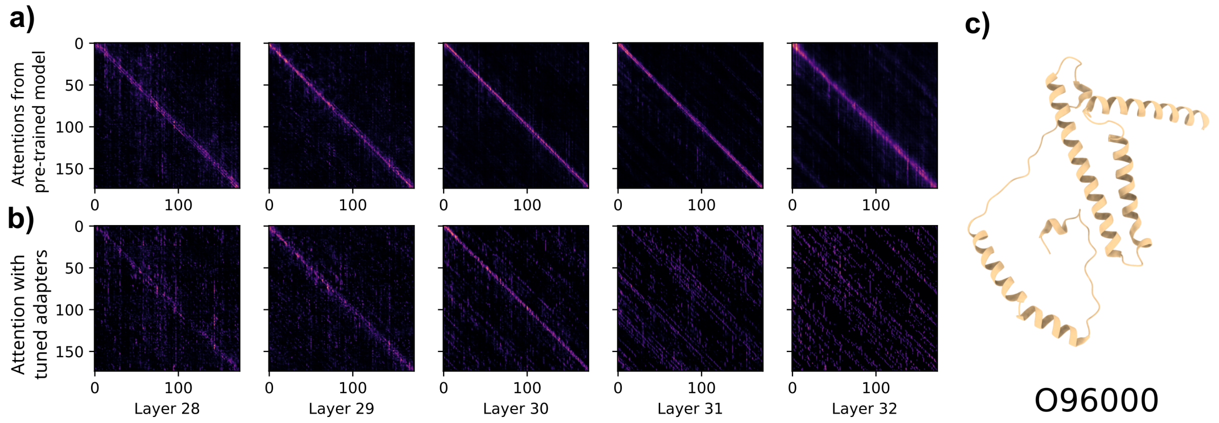

Fig. S5. Visualizing attention matrices. (a) Attentions for NADH dehydrogenase 1 β subcomplex subunit 10 (UniProt ID: O96000) using the pre-trained ESM2. **(b)** Attentions for the same protein after parameter-efficient fine-tuning. **(c)** Structure of O96000. We find that PEFT weights result in attention which is more spread out across the length of the protein when trained for PPI prediction.

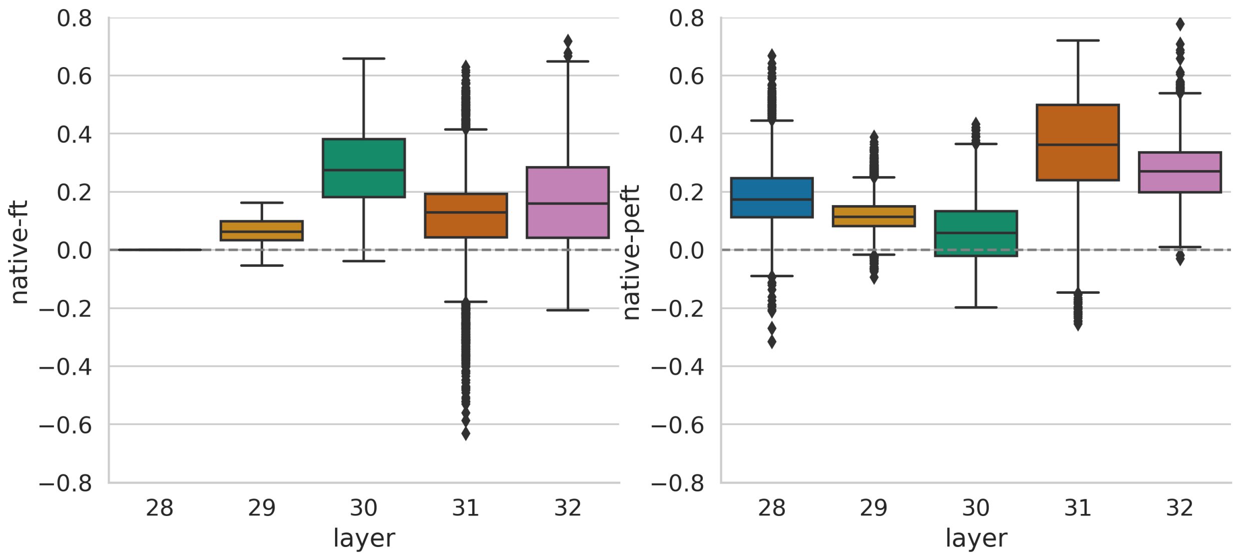

Fig. S6. We see similar increases in global attention in both traditional fine-tuning **(a)** and parameter-efficient fine-tuning **(b)**, when quantifying it with the diagonal correlation r_{xy}, described in detail in Equation 1.

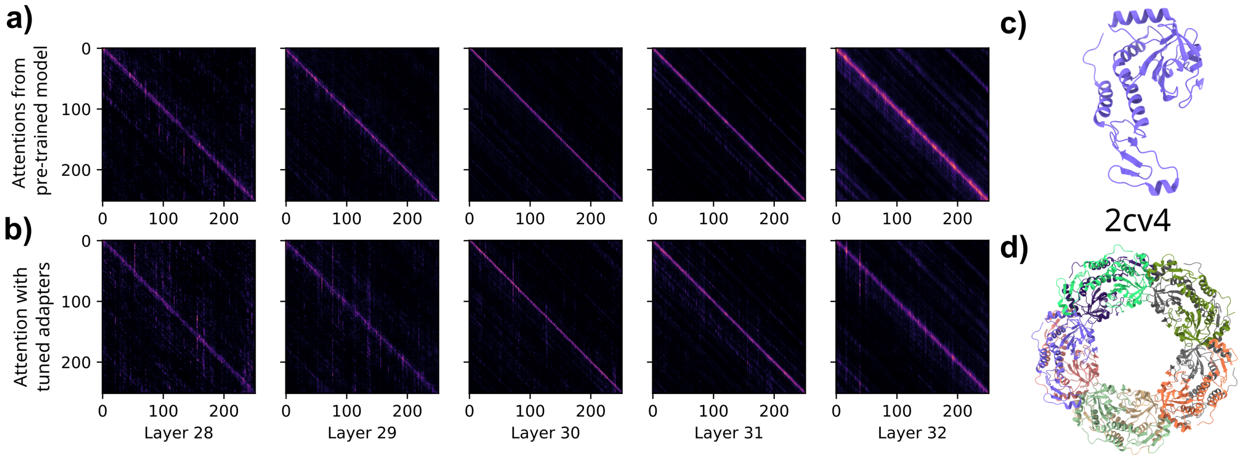

Fig. S7. Visualizing attention matrices. (a) Attentions for an Archael Peroxiredoxin from the Aerobic Hyperthermophilic Crenarchaeon Aeropyrum pernix K1 (PDB ID: 2CV4), which adopts a dihedral D5 symmetry, using the pre-trained ESM2. **(b)** Attentions for the same protein after parameter-efficient fine-tuning. **(c)** Structure of a single subunit 2CV4. **(d)** Structure of the 2CV4 homooligomer, with D5 symmetry. Here, find that PEFT weights result in attention which is only slight spread more spread across the length of the protein, but still remains concentrated near the diagonal.
